# Maternal reports of morbidity during the index ALSPAC pregnancy

**DOI:** 10.12688/wellcomeopenres.17900.2

**Published:** 2022-10-31

**Authors:** Genette Ellis, Abigail Fraser, Jean Golding, Yasmin Iles-Caven, Kate Northstone

**Affiliations:** 1 Bristol Medical School (Population Health Sciences), University of Bristol, Bristol, Bristol, BS8 2BN, UK

**Keywords:** ALSPAC, Pregnancy, Maternal health, Morbidity, Medications, Supplements

## Abstract

Within the ALSPAC (Avon Longitudinal Study of Parents and Children) resource, information concerning the health of the mother during pregnancy is available from three sources: (i) computerised data collected by midwives after the birth of the baby, known as the STORK database; (ii) data abstracted by ALSPAC staff from detailed medical obstetric records, and (iii) reports by mothers during pregnancy, and shortly after the birth using structured questionnaires completed at home. In this Data Note we focus on source (iii), and detail the information obtained from these mothers concerning their health, signs and symptoms together with medications and supplements taken during pregnancy. We also describe how the data can be accessed.

## Introduction

 Studies of mothers during pregnancy may use data from medical records or maternal report (the latter is often collected retrospectively sometime after the baby has been born). In general, prospective records are preferable to retrospective recall as they are more likely to be accurate. For this reason, the mothers enrolled in the Avon Longitudinal Study of Parents and Children (ALSPAC) were asked to complete four questionnaires spaced during the pregnancy, and a further questionnaire after the birth. Within three of these questionnaires were questions which asked about the health of the mother during defined periods of time, together with reasons for any medications taken.

In this document we outline the questions asked relating to the mother’s health and her signs and symptoms during the pregnancy. In addition, details of the reasons for the use of medications and the frequency with which she took analgesics such as paracetamol (acetaminophen) and aspirin. Elsewhere the actual medications the mother reported taking during pregnancy are detailed [ Headley *et al.*, 2004], as are details of other sources of information such as the data abstracted from the medical records [ Birmingham *et al.*, 2021] and the midwife recorded data on the STORK database [ Mummé *et al.*, 2020].

## Methods

 A consequence of enrolling women at unspecified times during pregnancy concerns the complexity of who received specific questionnaires at different time points. The strategy depended on the gestation of the pregnant mother at enrolment, described in detail elsewhere [ Iles-Caven *et al.*, 2020]. In brief, for mothers who had enrolled after 28 weeks gestation, and thus missed the first questionnaire ‘Your Environment’ a brief questionnaire named ‘Your Home and Lifestyle was sent later in pregnancy and the relevant data amalgamated together forming the A variables; at 18–23 weeks and 32+ weeks the time specific questionnaires Having a Baby and Your Pregnancywere administered. For those who had missed the 32+ week questionnaire a short questionnaire ‘Filling the Gaps’ was administered 12 months after the birth and the data that was not specific to the stage of pregnancy (e.g. education level, ethnic background) were amalgamated to form the C variables. Data from these questionnaires were given variable numbers each of which started with a letter of the alphabet: A (Your Environment/ *Your Home & Lifestyle*), B (Having a Baby/ *Your Home & Lifestyle*), C (Your Pregnancy/ *Filling the Gaps*), D (About Yourself) and E (Me and My Baby) respectively. Just three of these questionnaires were deliberately linked to a specific time period: B was administered at about 18 weeks gestation (it included health, signs, symptoms in first 3 months of pregnancy and from 4 months to time of questionnaire completion, feelings about becoming a parent, EPDS and CCEI, inter-personal sensitivity measure, activities and lifestyle); C at 32 weeks gestation (which included plans and expectations surrounding pregnancy and after the birth, health, signs and symptoms in previous 3 months, EPDS and CCEI, house moves in pregnancy, attitudes to infant feeding and care) and E at 8 weeks after birth (including health and lifestyle in late pregnancy). The other questionnaires (A and D) were administered at varying times according to the mother’s gestation at enrolment because the content was not time sensitive. For example, only 288/13,257 of Your Environment (A) were returned after the birth of the baby; if a mother enrolled after 31 weeks gestation, D (About Yourself) was administered at various stages of pregnancy and up to 4 months post-partum (1417/13,888 returned after the birth) [ Iles-Caven *et al.*, 2020]. At 8 weeks after birth, the mother completed a questionnaire about herself which covered the late stages of pregnancy as well as the period since birth (the E files).

In this descriptive paper we use the data from the B, C and E sources.

Please note that the study website contains details of all the data that is available through a fully searchable data dictionary and variable search tool.

### The ALSPAC sample

The study organisers invited pregnant women resident in a defined area of Avon, UK with expected dates of delivery between 1st April 1991 and 31st December 1992 to take part in the study. The initial number of pregnancies enrolled was 14,541, an estimated 80% of the eligible population ( Boyd *et al.*, 2013; Fraser *et al.*, 2013). 

For each item of data, we present in this paper the actual question used, the variable number, the number of pregnancies for which there are valid responses, and, where appropriate, the proportion responding positively. Variable numbers are in the format B040 where B is the questionnaire source (e.g. Having a Baby).

1. The mother’s health during pregnancy

*1.1. Subjective assessments of health*

The mothers were asked to rate their health at various stages from before pregnancy to the last 4 weeks of the pregnancy. The results ( Table 1.1) demonstrate that, on average, they felt least well in the first three months of pregnancy, but that the majority were feeling well and healthy subsequently.

**Table 1.1.** Mothers’ assessment of her own health before and during pregnancy.

Var No.	Time period	N	Always fit &well	Usually fit &well	Sometimes unwell	Often unwell	Always unwell
B040	Before pregnancy	12,007	31.9%	60.1%	7.0%	0.9%	0.1%
B041	First months of pregnancy	11,910	11.1%	29.2%	32.4%	20.0%	7.4%
B042	At 16 – 18 weeks gestation	11,131	26.1%	48.5%	19.7%	4.4%	1.2%
C050	At 30 – 32 weeks gestation	12,033	26.8%	48.2%	19.3%	4.7%	0.9%
E140	Last 4 weeks of pregnancy *	11,439	35.1%	50.9%	9.6%		4.4%

*Descriptors were changed to: Always fit and well; Mostly felt well and healthy; Often felt unwell; Hardly ever felt well

*1.2. Gastrointestinal signs and symptoms*

In Table 1.2 are shown the proportions of pregnant women who suffered from nausea, vomiting or diarrhoea at different stages of pregnancy. As expected, two-thirds of the mothers experienced nausea within the first 3 months of their pregnancy; proportionately fewer cases of vomiting (42%) were reported during this trimester, but the prevalence of diarrhoea increased during the second half of pregnancy. Although constipation is common in pregnancy, there were no direct questions on this, but the mothers were asked if they had taken any medication for the problem (see Section 2.1).

**Table 1.2.** Prevalence of nausea, vomiting and diarrhoea during pregnancy.

Var No.	Time period	N	Yes
	** Nausea**		
B044	First 3 months	12,130	68.5%
B043	4m – 18 weeks ^a^	12,130	13.2%
C052	18 – 32 weeks ^b^	12,054	36.7%
E100	32+ weeks ^c^	11,641	23.6%
	** Vomiting**		
B046	First 3 months	12,135	41.8%
B045	4m – 18 weeks ^a^	12,135	11.0%
C053	18 – 32 weeks ^b^	12,054	22.8%
E101	32+ weeks ^c^	11,641	12.2%
	** Diarrhoea**		
B048	First 3 months	12,135	16.5%
B047	4m – 18 weeks ^a^	12,135	7.8%
C054	18 – 32 weeks ^b^	12,054	29.6%
E102	32+ weeks ^c^	11,641	20.0%

^a ^After 3 months and by 18 weeks; ^b ^in the 3 months before 32 weeks; ^c ^from 7 months to the end of pregnancy.

*1.3. Infections*

The pregnant women were asked on four occasions whether they had had: (a) influenza; (b) rubella; (c) thrush (candida), (d) genital herpes, (e) urinary infection, or (f) any other infection. If the latter, they were asked to describe it. The text descriptions of these infections are available to interested researchers. Finally, a variable was derived to indicate, from the answers to these six questions, whether the women had had an infection of any sort during the pregnancy. The responses to all the questions on infection are listed in Table 1.3.

**Table 1.3.** Infections experienced by the women during pregnancy.

Var No.	Time period	N	Yes
	**Influenza**		
B056	First 3 months	12,135	8.4%
B055	4m – 18 weeks ^a^	12,135	5.7%
C059	18 – 32 weeks ^b^	12,054	5.9%
E106	32+ weeks ^c^	11,641	4.5%
	**Rubella**		
B058	First 3 months	12,136	<0.1%
B057	4m – 18 weeks ^a^	12,163	<0.1%
C060	18 – 32 weeks ^b^	12,054	<0.1%
E107	32+ weeks ^c^	11,641	<0.1%
	**Thrush**		
B060	First 3 months	12,133	8.9%
B059	4m – 18 weeks ^a^	12,133	5.6%
C061	18 – 32 weeks ^b^	12,054	13.8%
E108	32+ weeks ^c^	11,641	12.6%
	**Genital herpes**		
B062	First 3 months	12,136	0.2%
B061	4m – 18 weeks ^a^	12,136	0.1%
C062	18 – 32 weeks ^b^	12,054	0.4%
E109	32+ weeks ^c^	11,641	0.3%
	**Urinary infection**		
B054	First 3 months	12,134	4.7%
B053	4m – 18 weeks ^a^	12,134	2.6%
C057	18 – 32 weeks ^b^	12,054	6.4%
E105	32+ weeks ^c^	11,641	6.1%
	Another infection *		
B064	First 3 months	12,130	4.6%
B063	4m – 18 weeks ^a^	12,130	3.4%
C063	18 – 32 weeks ^b^	12,054	5.0%
E110	32+ weeks	11,639	3.5%
	**Any of above** ** infections**		
B065	First 3 months	12,225	22.9%
C064	18 – 32 weeks ^b^	12,054	26.3%
E111	32+ weeks ^c^	11,641	22.7%

^a^ After 3 months and by 18 weeks; ^b^ in the 3 months before 32 weeks; ^c^ from 7 months to the end of pregnancy. *Mother was asked to describe as text. This information can be requested from the ALSPAC Data Team.

*1.4. Depression and anxiety*

At two points during pregnancy (18- and 32-weeks gestation) the mothers’ depression and anxiety levels were measured using the following self-completion scales ( Table 1.4):

**Table 1.4.** Measures of mental health during pregnancy.

Measure	Var. no	Time period	N	Range
*Depression - EPDS*	B370	18 weeks	12,067	0–30
	B371	18 weeks	12,251	0–30
	C600	32 weeks	11,972	0–29
	C601	32 weeks	12,067	0–29
*Depression - CCEI*	B353	18 weeks	11,788	0–16
	B354a	18 weeks	12,139	0–16
	C579	32 weeks	11,630	0–16
	C580	32 weeks	11,947	0–16
*Somaticism - CCEI*	B355	18 weeks	11,921	0–14
	B356a	18 weeks	12,146	0–14
	C576	32 weeks	11,952	0–14
	C577	32 weeks	12,104	0–14
*Anxiety - CCEI*	B351	18 weeks	11,868	0–16
	B352a	18 weeks	12,140	0–16
	C579	32 weeks	11,656	0–16
	C580	32 weeks	11,950	0–16

(i) The Edinburgh Postnatal Depression Scale (EPDS) developed by Cox and colleagues (1987) is comprised of 10 items, specifically chosen by the authors because they did not involve somatic items. Each question had 4 response categories scored 0 to 3, which referred to the feelings of the mother in the past week. Higher scores on the EPDS are associated with more depressive symptoms. Although the measure was developed specifically for use with postnatal women, none of the 10 items is specific to the postnatal experience. The main feature of the scale that designates it as a postnatal scale is that it does not include somatic items because of the possibility of confounding somatic symptoms of depression with normal physiological symptoms at this time. Both our own pilot studies and the study of Murray and Carrothers (1990) found the measure to be acceptable to antenatal respondents, producing high completion rates with little evidence of response error. Validation of the scale during pregnancy, the post-partum period and early parenthood has been examined using standardised psychiatric interviews as the validating measures and it has been shown that the EPDS has high sensitivity and specificity (e.g. Thorpe, 1993). There are three variables relating to the EPDS score at both time points: the score derived from all 10 items completed (B370 and C600), the number of items with missing responses (B372 and C602), and the score derived by putting any missing item to the mode for that item unless all items were missing in which case the score was put to missing (B371 and C601).

The EPDS scores obtained in pregnancy have been used widely – both to demonstrate changes over time (e.g. Heron *et al.*, 2004) and associations with the development of the offspring (e.g. Deave *et al.*, 2008; Pearson *et al.*, 2013). Further details of the EPDS in ALSPAC are available elsewhere ( Paul & Pearson, 2020).

(ii) Three sub-scales of the Crown-Crisp Experiential Index (CCEI) relating to free-floating anxiety, depression and somaticism (8 questions in each) ( Crown & Crisp, 1979) were included in the questionnaires administered at 18 and at 32 weeks gestation (omitted were the sub-scales on phobia, obsession and hysteria). Although the total score of the 48 items in the original index has been shown to be a useful measure of psycho-neurotic pathology in the community, the need to limit the number of items and our specific interest in depression and anxiety guided the selection of these items. Indeed, most studies using the CCEI, including those of the original authors have focused on the sub-scales and it has been used in this way in the study of mental health of mothers during pregnancy and the postnatal year (e.g. O’Connor *et al*., 2002). The original three sub-scales had varying styles of response, some being a two-point yes/no scale, while others had 3-point categories (which had mixed options e.g. Very/Greatly/Frequently;Always/Often/Definitely; Fairly/A little/Sometimes; Not at all/Never/Not particularly). We kept the original questions with the lead in: ‘Please indicate the way you feel at this stage in your pregnancy’, but modified the response categories so that each item had four consistent response categories for the respondent to indicate the frequency of symptoms (Never/Not very often/Often/Very often)’. Score ranges were 0-16 for each of anxiety and depression and 0-14 for somaticism. CCEI total score range 0-44. These modifications were extensively pilot tested including a validation study against the Present State Examination ( Thorpe, 1993).

Maternal anxiety was measured using the relevant sub-scale of the CCEI, a validated self-rating inventory (e.g., Crisp *et al.*, 1978; example items include “worry a lot” and “feeling strung up inside”). In a pilot study of a random sample of 54 pregnant women attending a routine check-up, this score correlated 0.70 and 0.76 with the State and Trait (respectively) subscales of the Spielberger State-Trait Anxiety Inventory ( Blackmore *et al.*, 2011; Spielberger, 2010). There is no established cut-off for this measure. Some authors using this scale have identified mothers who scored in the top 15% (or as close as possible) as ‘very anxious’. The variables reporting the CCEI Anxiety score are: B351, B352b, B352a; C573, C575, C574 respectively. The number of these items where the data was missing is recorded; if all items were missing the score was put to missing, but for calculation of scores where only a few items were missing the mode has been substituted for the missing item.

Using the same strategy, the variables depicting the CCEI depression scale were: B353, B354B, B354A; C579, C581, C580; and those for the CCEI somatic scale were: B355, B356B, B356A; C576, C578, C577 for the scales using complete data; the numbers of missing items and the scales with missing items being put to the mode.

There have been a number of highly referenced ALSPAC publications using the Anxiety CCEI scale, showing the ways in which maternal anxiety varies over time ( Heron *et al.*, 2004) as well as ways in which it predicted early childhood behaviour (e.g. O’Connor *et al.*, 2002) as well as mood in late adolescence (e.g. Pearson *et al.*, 2013) in the offspring.

*1.5. Other specific signs and symptoms*

All other conditions for which there were specific questions are listed in Table 1.5. These comprise (a) vaginal bleeding; (b) jaundice; (c) an injury or shock; (d) glycosuria (sugar in the urine); (e) a cold; (f) headache; (g) backache; (h) varicose veins, and (i) other condition. If either items (c) or (i) were ticked, the mother was asked to describe the details – these can be retrieved, upon request, from the ALSPAC data team.

**Table 1.5.** Other specific signs and symptoms during pregnancy.

Var No.	Time period	N	Yes
	**Vaginal bleeding**		
B050	First 3 months	12,135	15.2%
B049	4m – 18 weeks ^a^	12,135	2.8%
C055	18 – 32 weeks ^b^	12,054	4.6%
E103	32+ weeks ^c^	11,641	4.6%
	**Jaundice**		
B052	First 3 months	12,138	0.1%
B051	4m – 18 weeks ^a^	12,138	<0.1%
C056	18 – 32 weeks ^b^	12,054	0.1%
E104	32+ weeks ^c^	11,641	0.1%
	Injury or shock *		
B068	First 3 months	12,140	4.8%
B067	4m – 18 weeks ^a^	12,140	3.5%
C066	18 – 32 weeks ^b^	12,054	7.5%
E112	32+ weeks ^c^	11,641	5.3%
	**Sugar in urine**		
B072	First 3 months	12,124	2.2%
	4m – 18 weeks ^a^	12,124	2.0%
C067	18 – 32 weeks ^b^	12,054	13.5%
E113	32+ weeks ^c^	11,641	20.5%
	**Had a cold**		
C058	18 – 32 weeks ^b^	12,054	40.5%
	**Had a headache**		
C073	18 – 32 weeks ^b^	12,054	61.4%
	**Had backache**		
C074	18 – 32 weeks ^b^	12,054	80.3%
	**Varicose veins**		
C075	18 – 32 weeks ^b^	12,054	14.9%
	Another problem *		
E116	32+ weeks ^c^	11,641	6.5%

^a^ After 3 months and by 18 weeks; ^b^ in the 3 months before 32 weeks; ^c^ from 7 months to the end of pregnancy.  *Mother was asked to describe as text. This information can be requested from the ALSPAC Data Team.

After the study pregnancies had been delivered it was realised that there were details concerning the types of early vaginal bleeding that it would be useful to collect. At 33 months after the birth the mother was therefore sent a questionnaire that included the following question: ‘During the first months of the study pregnancy, did you have any bleeding episodes?’ If she answered ‘yes’, she was asked to describe them, distinguishing between: spotting only/ one bleed a bit like a period / quite heavy bleeding/ other (please describe). The variables for these responses are H800 and H801. The text descriptions are available upon request from the ALSPAC data team.

2. Medications and supplements taken during pregnancy

As outlined in an earlier paper ( Headley *et al*., 2004), the mother was asked on four occasions (in files A, B, C and D) to list the medications (including ointments, herbal treatments and dietary supplements) that she had taken recently. These were all coded individually and are documented elsewhere ( Headley *et al.*, 2004). Here we document the medications for which there were direct questions and answers.

*2.1. Medications for specific conditions*

As shown in Table 2.1, questions were asked at 18 weeks concerning drugs taken during pregnancy, and at 32 weeks gestation regarding medications taken in the past three months. The conditions listed included nausea, heartburn, vomiting, anxiety, infection, migraine, sleep problems, pain, allergies, skin condition, bleeding, depression, haemorrhoids, constipation and cough. The mother was also asked to list any other problems for which she had taken medication during that time period. The list of such conditions is available from the ALSPAC data team.

**Table 2.1.** Medications taken by mother for specific conditions during pregnancy.

Var No.	Time period	N	Yes
	**Medication for Nausea**		
B101	First 3 months	13,047	4.5%
B100	4m – 18 weeks ^a^	13,456	1.0%
C090	18 – 32 weeks ^b^	11,943	2.3%
	**Medication for Heartburn**		
B103	First 3 months	13,026	6.5%
B102	4m – 18 weeks ^a^	13,026	9.8%
C091	18 – 32 weeks ^b^	11,943	36.5%
	**Medication for Vomiting**		
B105	First 3 months	13,045	3.6%
B104	4m – 18 weeks ^a^	13,045	1.1%
C092	18 – 32 weeks ^b^	11,943	1.8%
	**Medication for Anxiety**		
B107	First 3 months	13,045	0.5%
B104	4m – 18 weeks ^a^	13,045	0.2%
C093	18 – 32 weeks ^b^	11,943	0.7%
	**Medication for Infection**		
B107	First 3 months	13,037	8.1%
B108	4m – 18 weeks ^a^	13,037	6.0%
C094	18 – 32 weeks ^b^	11,943	11.5%
	**Medication for Migraine**		
B111	First 3 months	13,018	11.8%
B110	4m - 18 weeks ^a^	13,018	7.7%
C095	18 – 32 weeks ^b^	11,984	8.0%
	**Medication for Sleep problem**		
B113	First 3 months	13,038	0.8%
B112	4m – 18 weeks ^a^	13,038	1.0%
C096	18 – 32 weeks ^b^	11,943	3.3%
	**Medication for Pain**		
B115	First 3 months	13,023	11.5%
B114	4m - 18 weeks ^a^	13,023	9.4%
C097	18 – 32 weeks ^b^	11,943	15.7%
	**Medication for Allergy**		
B117	First 3 months	13,035	2.6%
B116	4m – 18 weeks ^a^	13,035	2.2%
C098	18 – 32 weeks ^b^	11,943	4.0%
	**Medication for Skin condition**		
B119	First 3 months	13,029	6.8%
B118	4m – 18 weeks ^a^	13,029	5.8%
C099	18 – 32 weeks ^b^	11,943	11.1%
	**Medication for Bleeding**		
B121	First 3 months	13,041	0.5%
B120	4m – 18 weeks ^a^	13,041	0.2%
C100	18 – 32 weeks ^b^	11,943	0.4%
	**Medication for Depression**		
B123	First 3 months	13,044	0.6%
B122	4m – 18 weeks ^a^	13,044	0.5%
C101	18 – 32 weeks ^b^	11,943	0.9%
	**Medication for Haemorrhoids**		
B125	First 3 months	13,036	2.1%
B124	4m – 18 weeks ^a^	13,036	2.4%
C102	18 – 32 weeks ^b^	11,943	7.6%
	**Medication for Constipation**		
B127	First 3 months	13,029	5.2%
B126	4m – 18 weeks ^a^	13,029	3.7%
C103	18 – 32 weeks ^b^	11,943	7.1%
	**Medication for Cough**		
B129	First 3 months	13,030	5.2%
B128	4m – 18 weeks ^a^	13,030	4.6%
C104	18 – 32 weeks ^b^	11,943	7.8%
	Medication for Other reason *		
B131	First 3 months	12,888	6.3%
B130	4m – 18 weeks ^a^	13,044	5.2%
C105	18 – 32 weeks ^b^	11,943	9.7%

^a^After 3 months and by 18 weeks; ^b^in the 3 months before 32 weeks; ^c^from 7 months to the end of pregnancy; *Text descriptions available

*2.2. Frequency of taking analgesics, sleeping pills and tranquillisers*

Although the frequency with which medication was taken was not obtained for the medications in Table 2.1, specific questions on frequency were asked of paracetamol (acetaminophen), aspirin, Anadin or codeine, sleeping tablets and tranquillisers. Far more study mothers were reporting having taken paracetamol at some time during the pregnancy than any other form of medication: 55% by 18 weeks and 44% in the 3 months prior to 32 weeks gestation ( Table 2.2). Consequently, the data available to analyse the possible long-term effects of fetal exposure to this drug is more statistically powerful than for any of the other medications. Results of different analyses using these data have shown associations between maternal paracetamol exposure and asthma in the exposed offspring ( Shaheen *et al.*, 2010) as well as with early neurocognitive abnormalities ( Golding *et al.*, 2020).

**Table 2.2.** Frequency analgesics, sleeping tablets or tranquillisers used prior to: (a) 18 weeks gestation (b) 32 weeks gestation.

Var No.	Medicine	N	Yes, daily	Yes, most Days	Yes, sometimes	All Yes *	Never
	*Before 18 weeks*						
B170	Aspirin	13,074	0.6%	0.1%	4.2%	4.8%	95.2%
B171	Paracetamol	13,049	0.2%	1.3%	53.0%	54.5%	45.5%
B172	Codeine or Anadin	13,070	-	-	2.5%	2.5%	97.5%
B173	Sleeping tablets	13,072	-	-	-	0.5%	99.5%
B174	Tranquilisers	13,069	0.1%	-	0.2%	0.3%	99.7%
	*18- 32 weeks*						
C130	Aspirin	11,988	0.6%	0.1%	2.6%	3.3%	96.7
C131	Paracetamol	11,988	0.2%	0.9%	42.8%	43.9%	56.1%
C132	Codeine or Anadin	11,988	0.1%	0.1%	1.9%	2.0%	98.0%
C133	Sleeping tablets	11,988	<0.1%	0.1%	0.7%	0.7%	99.3%

*All yes = sum of ‘Yes, daily’, ‘Yes, most days’ and ‘Yes, sometimes’

It should be noted that the Bristol Obstetric Departments were involved in a randomised controlled trial ( CLASP, 1994) designed to assess whether low-dose aspirin in pregnancy would reduce the risk of pre-eclampsia. Obviously, the women who were taking part in the trial were not aware whether they were taking the placebo or aspirin and will have been recorded as taking aspirin. We suggest that these participants should be omitted from any analysis of the consequences of taking aspirin using the variable MZ052.

*2.3. Complementary medications*

From the list of medications and treatments listed in the various questionnaires, an exercise was undertaken to classify those that were considered to be “Complementary and Alternative Medicines” (CAMs). These were largely classified as herbal or homeopathic substances and are described elsewhere ( Bishop *et al.*, 2011). A separate question in the 18- and 32-week questionnaires asked for the frequency with which the mother had ever used homeopathic medications. The results are shown in Table 2.3.

**Table 2.3.** Frequency mother has ever used homeopathic medications as asked at 18- and 32-weeks gestation.

Var No.	N	Yes, often	Yes, sometimes	No
B160	12,985	0.9%	8.9%	90.2%
C120	12,297	0.8%	13.9%	85.3%a

*2.4. Dietary and mineral supplements*

As well as listing the dietary and mineral supplements taken, the mother completed specific questions concerning whether she had taken, in the preceding 3 months, the mineral supplements of iron, zinc or calcium, dietary supplements containing vitamins, folate or folic acid, or other types of supplement or diet food ( Table 2.4). Those who ticked ‘vitamins’ or ‘other types of supplement’ were asked to describe them. These data are available as text from the ALSPAC data team.

**Table 2.4.** Vitamin and mineral supplements taken in the previous 3 months.

Var No.	Vitamin or Mineral	N	Yes
	***Prior to 18 weeks***		
B140	Iron	13,030	22.5%
B141	Zinc	13,028	1.3%
B142	Calcium	13,021	3.5%
B143	Folic Acid	13,016	9.0%
B144	“Vitamins”	12,988	16.4%
B149	Other supplements or diet food	12,389	2.8%
	***Prior to 32 weeks***		
C110	Iron	12,006	43.1%
C111	Zinc	12,006	1.3%
C112	Calcium	12,006	3.6%
C113	Folic Acid	12,006	18.4%
C114	“Vitamins”	12,006	11.6%
C115	Other supplements or diet food	12,006	2.3%

*2.5. Numbers of different medications and/or supplements taken*

From the lists of different medications and supplements listed, the data team computed the actual numbers of different substances taken in the preceding 3 months. The numbers recorded at 18 weeks ranged from 0 to 17 [Variable B180], and at 32 weeks from 0 to 31 [C135]. As mentioned above in 2.2. it is important to note that a subgroup of the pregnant women (n=42) were taking part in a randomised controlled trial of low dose aspirin known as the CLASP study. They can be identified using variable MZ052.

## 3. Procedures reported

### 3.1. Medical tests and procedures

Details of the gestation at which various tests and procedures were reported by the mother as being undertaken are given in Table 3.1 for X-rays, amniocentesis, CVS (chorionic villous sampling), AFP (alpha-feto protein), ultrasound examinations and hospital admissions. The latter exclude admissions after the onset of labour. It should be noted that these details are also recorded in the dataset of information extracted from the medical records ( Birmingham *et al.*, 2021). 

**Table 3.1.** Tests and procedures experienced by the women during pregnancy prior to labour and the birth.

Var No.	Time period	N	Yes
	**X-ray**		
B074	First 3 months	12,213	1.9%
B073	34m – 18 weeks ^a^	12,214	4.0%
C068	18 – 32 weeks ^b^	12,090	1.1%
E114	32+ weeks ^c^	11,639	2.9%
	**Amniocentesis**		
B076	First 3 months	12,209	0.5%
B075	3m – 18 weeks ^a^	12,209	3.3%
C069	18 – 32 weeks ^b^	12,090	1.6%
	**CVS**		
B078	First 3 months	12,207	0.9%
B077	3m – 18 weeks ^a^	12,207	1.6%
C070	18 – 32 weeks ^b^	12,090	0.7%
	**AFP test**		
B080	First 3 months	12,207	13.0%
B079	3m – 18 weeks ^a^	12,207	74.7%
C071	18 – 32 weeks ^b^	12,090	22.5%
	**Ultrasound** **scan**		
B082	First 3 months	12,200	27.4%
B081	3m – 18 weeks ^a^	12,200	80.7%
C072	18 – 32 weeks ^b^	12,090	44.3%
E115	32+ weeks ^c^	11,639	34.2%
	**Hospital admission**		
B083	0 – 18 weeks ^a^	12,157	3.9%
C076	18 – 32 weeks ^b^	12,025	7.4%
E120	7m – before labour	11,577	27.0%

*AFP = alpha-fetoprotein; CVS = *Chorionic villus sampling*.

### 3.2. Dental procedures

Information on exposure to dental procedures occurring during pregnancy were asked on two occasions, both retrospectively: (a) at 8 weeks after the birth the mother was asked about attendance at a dentist, how many fillings were given and the month of pregnancy at which the first filling was given (E – Me and My Baby); (b) subsequently it was realised that further information on dental amalgams, dental X-rays, and dental anaesthetic could be valuable - but this information was not collected until 33 months after the birth (H -Your Health, Events & Feelings) ( Table 3.2). Nevertheless, the information on dental amalgam was shown to be associated with maternal blood mercury level, thus providing some validation of these retrospective measures ( Golding *et al.,* 2016).

**Table 3.2.** Information on dental treatment during pregnancy.

Var no.	Dental information	N	Results
	*Recollections 8 weeks after the birth (E – Me and My Baby)*		
E090	Visited a dentist during pregnancy	5,479	75.5% visited dentist
E091	No. of fillings inserted	5,436	Range 0–9 20% had at least 1 filling
E092	Month of pregnancy when had first filling	1,823	Range 0–9; mode = 4 months
	*Recollections 33 months (H – Your Health, Events & Feelings) after the birth*		
H790	No. of amalgam fillings at start of pregnancy *	8,643	None: 7.8%; one: 5.7%; 2–3: 6.8%; 4+: 69.8%.
H791	Visited dentist during pregnancy	9,561	86.1 yes; 11.6% no; 2.3% unsure
H792	Had teeth extracted	9,413	3.8% yes
H793	Had dental amalgam fillings inserted	9,413	24.1% yes
H794	Had dental amalgam fillings extracted	9,413	15.3% yes
H795	Had dental gas (as anaesthetic)	9,413	0.5% yes
H796	Had dental X-ray	9,413	7.8% yes
H797	No. of dental X-rays	9,384	Range 0–5

*See text for the question asked

## Strengths and limitations of the data

The participants recruited to the study were broadly representative of the general population of new parents resident in the area at the time in terms of sex, ethnicity and socio-economic status ( Fraser *et al.*, 2013). All mothers who answered at least one of the four early questionnaires (A,B,C,D) and had a record of the pregnancy outcome were included in the descriptions in this paper. The data are linkable to all other data collected throughout the study. This includes information about the relationships with partners and the study child, biological markers from different members of the family, data regarding the parents’ beliefs and behaviours, physical and psychological environments, life experiences and demographics. The advantage of collecting information prospectively on common symptoms as well as over the counter medications is that such information is unlikely to be found in medical records or even recalled months or years later.

A limitation of this study is the lack of diversity, because at the time of enrolment, the county of Avon was mainly Caucasian, therefore there were too few Black, Asian and Minority Ethnic (BAME) participants (<6%) to allow for detailed analysis by ethnic background.

In addition, as with all longitudinal studies, there is a loss to follow up over time, either through participants moving and failing to notify the study, dying, or rarely, withdrawing their consent for the study. In the latter case the data are removed. Consequently, the numbers involved in any analyses using these data will be slightly less than shown in this paper.

## Data Availability

ALSPAC data access is through a system of managed open access. The steps below highlight how to apply for access to the data included in this paper and all other ALSPAC data. Please read the ALSPAC access policy ( http://www.bristol.ac.uk/media-library/sites/alspac/documents/researchers/data-access/ALSPAC_Access_Policy.pdf) which describes the process of accessing the data and biological samples in detail, and outlines the costs associated with doing so. You may also find it useful to browse our fully searchable research proposals database ( https://proposals.epi.bristol.ac.uk/), which lists all research projects that have been approved since April 2011. Please submit your research proposal ( https://proposals.epi.bristol.ac.uk/) for consideration by the ALSPAC Executive Committee using the online process. You will receive a response within 10 working days to advise you whether your proposal has been approved. If you have any questions about accessing data, please email: alspac-data@bristol.ac.uk (data) or bbl-info@bristol.ac.uk (samples).
