# Supplementary material for: Maternal reports of morbidity during the index ALSPAC pregnancy
Source: Wellcome Open Res. 2022 Oct 31;7:163. Originally published 2022 May 26. [Version 2] doi: 10.12688/wellcomeopenres.17900.2 (PMC9699990; doi:10.12688/wellcomeopenres.17900.2)
Supplement: Peer review history

# Peer review: Reviewer response for version 2

**DOI:** 10.21956/wellcomeopenres.20527.r53071

**Published:** 2022-11-25

**Authors:** Deborah Dewey

**Affiliations:** 1 Alberta Children's Hospital Research Institute, University of Calgary, Calgary, AB, Canada; 2 Department of Pediatrics, Cumming School of Medicine, University of Calgary, Calgary, AB, Canada; 3 Department of Community Health Sciences, Cumming School of Medicine, University of Calgary, Calgary, AB, Canada

I have two minor comments for the authors to address. Once these are addressed, I approve the article.

- The following sentence is too long and complicated and very difficult to follow. The authors should consider revising it. They may want to discuss the time specific questionnaires first and then the questionnaires that mothers who enrolled after 28 weeks gestation completed.
*“In brief, for mothers who had enrolled after 28 weeks gestation, and thus missed the first questionnaire ‘Your Environment’ a brief questionnaire named ‘Your Home and Lifestyle was sent later in pregnancy and the relevant data amalgamated together forming the A variables; at 18–23 weeks and 32+ weeks the time specific questionnaires Having a Baby and Your Pregnancy were administered.”*
- In the following sentence the authors talk about variable numbers; however, no numbers were used. Variable letters were used. This should be changed.
*“Data from these questionnaires were given variable numbers each of which started with a letter of the alphabet: A (Your Environment/Your Home & Lifestyle), B (Having a Baby/Your Home & Lifestyle), C (Your Pregnancy/Filling the Gaps), D (About Yourself) and E (Me and My Baby) respectively.”*

Are sufficient details of methods and materials provided to allow replication by others?

Partly

Is the rationale for creating the dataset(s) clearly described?

Yes

Are the datasets clearly presented in a useable and accessible format?

Yes

Are the protocols appropriate and is the work technically sound?

Yes

Reviewer Expertise:

NA

I confirm that I have read this submission and believe that I have an appropriate level of expertise to confirm that it is of an acceptable scientific standard.

# Peer review: Reviewer response for version 1

**DOI:** 10.21956/wellcomeopenres.19835.r52559

**Published:** 2022-10-14

**Authors:** Deborah Dewey

**Affiliations:** 1 Alberta Children's Hospital Research Institute, University of Calgary, Calgary, AB, Canada; 2 Department of Pediatrics, Cumming School of Medicine, University of Calgary, Calgary, AB, Canada; 3 Department of Community Health Sciences, Cumming School of Medicine, University of Calgary, Calgary, AB, Canada

This paper provides a summary of the data that is available from ALSAP regarding maternal reports of health and signs and symptoms during the index pregnancy. In addition, data on reasons for medication use and the frequency of use of analgesics is provided as are data on medical and dental procedures.

 The information on data collection methods was not clear and more detail is required. There also appeared to be some inconsistencies between the methods reported in this report and those reported in the previously referenced study by Iles-Carven et al. (2020). There are also some statements made by the authors that would benefit from references.

 The data tables and information as to how the data can be accessed is clear.

 The following comments are provided for the authors’ consideration:

1. In the Methods section of this paper it states:
  “ *In brief, data from these five questionnaires were given variable numbers each of which started with a letter of the alphabet: A, B, C, D and E respectively*”.
  What five questionnaires are being referred to? Please expand on this in the present Data Note so the reader knows what questionnaires are being referred to.
  In the Iles-Carven paper the questionnaires listed are:
  - Your Environment (A)
  - Having a Baby (B)
  - Your Pregnancy (C)
  - About Yourself (D)
  - Your Home Life and Filling Gaps (E)
  What are the authors referring to by the term “variable numbers”? Do the authors mean that the five questionnaires were labelled A, B, C, D, and E? How are A, B, C, D and E related to variable numbers? This is not clear to the reader.
2. In the Methods, it also states the following:
  “ *Just three of these five questionnaires were deliberately linked to a specific gestational period: B was administered at about 18 weeks’ gestation; C at 32 weeks’ gestation and E at 8 weeks post-delivery*.”
  It seems odd to suggest that 8 weeks post-delivery is a gestational period.  I would suggest the authors re-phrase this sentence. It would be helpful if the authors clearly indicated what specific gestational period each of the questionnaires was focused on. For example, was questionnaire E focused on the period between >32 weeks’ gestation and childbirth?
3. It is important to be consistent among the papers published on the ALSPAC cohort. The Iles-Carven et al  (2020) paper it states that the postnatal questionnaire was sent out at 12 weeks post-delivery. Here in this paper the authors state that the questionnaire was sent out at 8 weeks post-delivery. When was it sent out? Please clarify and ensure that these paper and the Iles-Carven paper report the same information.
4. “ *The other questionnaires (A and D) were administered at variable times according to the mother’s gestation at enrolment*” 
  The Iles-Carven et al (2020) paper it states that 4 questionnaires were administered during pregnancy. Could the authors please clarify that A and D were administered during the mother’s pregnancy and ensure that this information is included in the Methods?
5. The authors state the following:
  “ *Three of the questionnaires were deliberately linked to a specific gestational period*”
  It is no clear to this reviewer, why the term “deliberately” is used. I would suggest that the authors provide some explanation as to why these questionnaires were deliberately linked to specific gestational time periods. Was the post-delivery questionnaire at 8 or 12 weeks post-delivery focused on a specific gestational time period? Did it ask any questions related to the first three months post-delivery?
6. In the section “The ASPAC sample second paragraph, I believe that comma after “and” should be removed. It should read as follows:
  “ *there are valid responses, and where appropriate, the ….*”  
7. In the section Depression and Anxiety I would suggest the following re-wording:
  " *The Edinburgh Post-Natal………. (1987) is comprised of 10 items and was specifically chosen by the authors because it did not include somatic items.*"
8. In the same section I would suggest adding a comma as follows:
  " *4 response categories scored 0 to 3 **, **which………*"
9. The authors’ need to include a statement indicating that higher scores on the EDPS were associated with more depressive symptoms.
10. This reviewer would suggest re-wording this sentence (bottom of page 4) as follows: 
    
  Validation of the scale during pregnancy, the post-partum period and early parenthood has been examined using standardised psychiatric interviews as the validating measures and it has been shown that the EPDS has high sensitivity and specificity. 
11. On page 5 the authors state “ *it has been used in this way in the study of mental health of mothers during pregnancy and the post-natal year.*” Please clarify the study being referred to by providing a reference.
12. What were the 3-point category Reponses of the original CCEI? Please provide this information
13. More detail needs to be provided regarding the Crown-Crisp Experiential Index as this measure is less familiar to most readings. In the description of all three subscales (i.e., anxiety, depression, and somatic and their adaptation for this study please clarify how many items are included in each subscale? What responses women could provide; were they yes-no or did they respond on a rating scale? If a rating scale was used what were the responses? What were the ranges of scores participants could obtain on these scales?
14. The authors state when discussing the DDEI *“internal consistencies (α) exceeded 0.80 for each of the four assessments*”. What four assessments are being referred to here? Please clarify.
15. Please change the ; after measure to ' *a*' .
  “ *There is no established cut-off for this measure*;”
16. Please provide references to support the following statement.
*“Some authors using this scale have identified mothers who scored in the top 15% (or as close as possible) as ‘very anxious."*
17. Please clarify that is meant by the statement “ *using variable excluding those with missing values*”. More detail is required.
18. This is not a complete sentence. The authors need to re-word this statement so that it is clear to the reader.
  “ *The number of missing items, and those where the mode has been substituted for a missing item (if, however, all items were missing then the latter variable was put to missing).*"
19. On page 6 the authors’ state:
  “ *As outlined in an earlier paper, the mother was asked on four occasions to list the medications (including ointments, herbal treatments and dietary supplements)*”
  What earlier paper? Please provide the reference/ What four occasions? Please list the specific questionnaires or time points?
20. On page 8 was the Dental procedures information collected at 8 weeks post-delivery or 12 weeks? When was the post-delivery questionnaire information collected at 8 or 12 weeks post-delivery?
21. What are the early questionnaires (A, B, C, and D?????) Please clarify.

Are sufficient details of methods and materials provided to allow replication by others?

Partly

Is the rationale for creating the dataset(s) clearly described?

Yes

Are the datasets clearly presented in a useable and accessible format?

Yes

Are the protocols appropriate and is the work technically sound?

Yes

Reviewer Expertise:

Maternal and child health research; longitudinal cohort studies, neurodevelopment

I confirm that I have read this submission and believe that I have an appropriate level of expertise to confirm that it is of an acceptable scientific standard, however I have significant reservations, as outlined above.

# Response

**Published:** 2022-10-24

**Authors:** Yasmin Iles-Caven

**Affiliations:** University of Bristol, UK

We are very grateful to the reviewer for their very helpful comments which have enabled us to clarify various aspects of the data collection description. We hope the paper is now much clearer.  Responses to each comment are in bold.

 "The following comments are provided for the authors’ consideration:

 In the Methods section of this paper it states:

 “ *In brief, data from these five questionnaires were given variable numbers each of which started with a letter of the alphabet: A, B, C, D and E respectively*”.

 What five questionnaires are being referred to? Please expand on this in the present Data Note so the reader knows what questionnaires are being referred to. **We have done this**

 In the Iles-Caven paper the questionnaires listed are:

 - Your Environment (A)

 - Having a Baby (B)

 - Your Pregnancy (C)

 - About Yourself (D)

 - Your Home Life and Filling Gaps (E) **E was the postnatal questionnaire Me and My Baby.  We have substantially rewritten the Methods and hope this is now clarified.**

 What are the authors referring to by the term “variable numbers”? Do the authors mean that the five questionnaires were labelled A, B, C, D, and E? How are A, B, C, D and E related to variable numbers? This is not clear to the reader. **Clarified **

1. In the Methods, it also states the following:
  “ *Just three of these five questionnaires were deliberately linked to a specific gestational period: B was administered at about 18 weeks’ gestation; C at 32 weeks’ gestation and E at 8 weeks post-delivery*.”
  It seems odd to suggest that 8 weeks post-delivery is a gestational period.  I would suggest the authors re-phrase this sentence. It would be helpful if the authors clearly indicated what specific gestational period each of the questionnaires was focused on. For example, was questionnaire E focused on the period between >32 weeks’ gestation and childbirth? **We have changed the wording to reflect the input of the E questionnaire.**
2. It is important to be consistent among the papers published on the ALSPAC cohort. The Iles-Carven et al  (2020) paper it states that the postnatal questionnaire was sent out at 12 weeks months post-delivery [ Filling the Gaps]. Here in this paper the authors state that the questionnaire was sent out at 8 weeks post-delivery. When was it sent out? Please clarify and ensure that these paper and the Iles-Carven paper report the same information.   We apologise for the confusion over when the different questionnaires were sent out. **The description of these has now been rewritten and clarified. **
3. “ *The other questionnaires (A and D) were administered at variable times according to the mother’s gestation at enrolment*” 
  The Iles-Carven et al (2020) paper it states that 4 questionnaires were administered during pregnancy. Could the authors please clarify that A and D were administered during the mother’s pregnancy and ensure that this information is included in the Methods? **We hope this is now clarified**
4. The authors state the following:
*Three of the questionnaires were deliberately linked to a specific gestational period*”
  It is no clear to this reviewer, why the term “deliberately” is used. I would suggest that the authors provide some explanation as to why these questionnaires were deliberately linked to specific gestational time periods. Was the post-delivery questionnaire at 8 or 12 weeks post-delivery focused on a specific gestational time period? Did it ask any questions related to the first three months post-delivery? **We hope this has been clarified now**
5. In the section “The ASPAC sample second paragraph, I believe that comma after “and” should be removed. It should read as follows:
  “ *there are valid responses, and where appropriate, the ….*” **Done**
6. In the section Depression and Anxiety I would suggest the following re-wording:
*The Edinburgh Post-Natal………. (1987) is comprised of 10 items and was specifically chosen by the authors because it did not include somatic items.*" **We have reworded as suggested**
7. In the same section I would suggest adding a comma as follows:
  " *4 response categories scored 0 to 3 **, **which………*" **Done**
8. The authors’ need to include a statement indicating that higher scores on the EDPS were associated with more depressive symptoms. **We have added this**.
9. This reviewer would suggest re-wording this sentence (bottom of page 4) as follows:  
   Validation of the scale during pregnancy, the post-partum period and early parenthood has been examined using standardised psychiatric interviews as the validating measures and it has been shown that the EPDS has high sensitivity and specificity. **We have reworded as suggested.**
10. On page 5 the authors state “ *it has been used in this way in the study of mental health of mothers during pregnancy and the post-natal year.*” Please clarify the study being referred to by providing a reference. **Done**
11. What were the 3-point category Reponses of the original CCEI? Please provide this information   
12. More detail needs to be provided regarding the Crown-Crisp Experiential Index as this measure is less familiar to most readings. In the description of all three subscales (i.e., anxiety, depression, and somatic and their adaptation for this study please clarify how many items are included in each subscale? What responses women could provide; were they yes-no or did they respond on a rating scale? If a rating scale was used what were the responses? What were the ranges of scores participants could obtain on these scales? **The authors have expanded on the description of the CCEI as requested and hope this is much clearer.**
13. The authors state when discussing the CCEI *“internal consistencies (α) exceeded 0.80 for each of the four assessments*”. What four assessments are being referred to here? Please clarify. **We agree that this was confusing and consequently has now been omitted** 
14. Please change the ; after measure to ' *a*' . **Done**
  “ *There is no established cut-off for this measure*;”
15. Please provide references to support the following statement.
*“Some authors using this scale have identified mothers who scored in the top 15% (or as close as possible) as ‘very anxious."* ***O’Connor et al 2002 has been added here.***
16. Please clarify that is meant by the statement “ *using variable excluding those with missing values*”. More detail is required. **We hope this has been clarified**
17. This is not a complete sentence. The authors need to re-word this statement so that it is clear to the reader.
  “ *The number of missing items, and those where the mode has been substituted for a missing item (if, however, all items were missing then the latter variable was put to missing).*" **This has been rewritten**
18. On page 6 the authors’ state:
  “ *As outlined in an earlier paper, the mother was asked on four occasions to list the medications (including ointments, herbal treatments and dietary supplements)*”
  What earlier paper? Please provide the reference/ What four occasions? Please list the specific questionnaires or time points? **Again, this has been clarified and a reference added.**
19. On page 8 was the Dental procedures information collected at 8 weeks post-delivery or 12 weeks? When was the post-delivery questionnaire information collected at 8 or 12 weeks post-delivery? **We confirm that this was at 8 weeks and have clarified.**

What are the early questionnaires (A, B, C, and D?????) Please clarify. . **This is correct and has been clarified in the text.**

# Peer review: Reviewer response for version 1

**DOI:** 10.21956/wellcomeopenres.19835.r50888

**Published:** 2022-07-05

**Authors:** Rachel Rowe

**Affiliations:** 1 National Perinatal Epidemiology Unit, Nuffield Department of Population Health, University of Oxford, Oxford, UK

This paper is a helpful summary of the data available within the ALSPAC resource about the physical and mental health of women during pregnancy collected from women themselves, largely prospectively, during pregnancy and shortly after birth. Data about medications, medical tests, and medical and dental procedures are also described. 

 The rationale for data collection is detailed in full elsewhere in the ALSPAC gateway. Data collection methods are clearly described and are technically sound. Data are summarised clearly and information about how to access the data is clear.

 I have a number of very minor comments:

1.  In Table 1.1 'Months' should not have a capital M and 'at' should have a capital A
2. In Table 1.2 the period described in the footnote as 'in the 3 months before 32 weeks' is described as '18-32 weeks'. In Table 1.3 this same period is described as '20-32 weeks'. I assume the latter is correct, but it's not clear
3. In Table 1.3 no data are presented for 'Another infection' at 32+weeks - are these missing? For 'Any infection', '1st 3 months' should be written as 'First 3 months' for consistency, and data for 4m-18 weeks appear to be missing.
4. Use postnatal or post-natal consistently.
5. In Table 2.1 it is not clear from the text why there are data for 32+ weeks for medication for vomiting only. Are data for this period for other medications not available or are they missing from the table?
6. In Table 2.2, one of the response options listed is 'All Yes'. It wasn't immediately clear to me what this means, but I see now that it simply means that the woman reported taking that medication at some point (either daily, most days, or sometimes). Can a footnote be added to explain this?
7. In Table 3.1 the footnotes for superscripts a, b, and c are missing. It is not clear to me why the time periods for the various tests and procedures are different. Can this be explained please?
8. Not essential, but it would be my preference to use the word 'birth' instead of 'delivery' where possible.

Are sufficient details of methods and materials provided to allow replication by others?

Yes

Is the rationale for creating the dataset(s) clearly described?

Yes

Are the datasets clearly presented in a useable and accessible format?

Yes

Are the protocols appropriate and is the work technically sound?

Yes

Reviewer Expertise:

Maternity services research

I confirm that I have read this submission and believe that I have an appropriate level of expertise to confirm that it is of an acceptable scientific standard.

# Response

**Published:** 2022-10-24

**Authors:** Yasmin Iles-Caven

**Affiliations:** University of Bristol, UK

We thank the reviewer for their very helpful comments which have enabled us to clarify certain aspects of the data collection.

 I have a number of very minor comments:

1.  In Table 1.1 'Months' should not have a capital M and 'at' should have a capital A - **We have corrected this.**
2. In Table 1.2 the period described in the footnote as 'in the 3 months before 32 weeks' is described as '18-32 weeks'. In Table 1.3 this same period is described as '20-32 weeks'. I assume the latter is correct, but it's not clear. **This has been corrected to 18-32 weeks throughout **
3. In Table 1.3 no data are presented for 'Another infection' at 32+weeks - are these missing? **This has now been inserted in the table**. For 'Any infection', '1st 3 months' should be written as 'First 3 months' ( **done**) for consistency,
4. For ‘any infection’ data for 4m-18 weeks appear to be missing. **This has been clarified to ‘Any of the above infections’ **
5. Use postnatal or post-natal consistently. **We have done this.**
6. In Table 2.1 it is not clear from the text why there are data for 32+ weeks for medication for vomiting only. Are data for this period for other medications not available or are they missing from the table? **Apologies, vomiting medication was a typo and has been omitted. **       
7. In Table 2.2, one of the response options listed is 'All Yes'. It wasn't immediately clear to me what this means, but I see now that it simply means that the woman reported taking that medication at some point (either daily, most days, or sometimes). Can a footnote be added to explain this? **We have clarified this.**
8. In Table 3.1 the footnotes for superscripts a, b, and c are missing. It is not clear to me why the time periods for the various tests and procedures are different. Can this be explained please? **We have corrected this.**
9. Not essential, but it would be my preference to use the word 'birth' instead of 'delivery' where possible. **We have altered where appropriate and think this reads better. **
